# Cancer Research

**Published:** 1902-08-02

**Authors:** 


### The Hospital

A Journal of

The Medical Sciences and Hospital Administration.

Vol. XXXII.—No. 827. Edited by Sir Henry Burdett, K.C.B., and Solomon C. Smith, M.D., M.R.C.P. [August 2, 1902.

### Cancer Research.

The possibility of fruitfully conducting a sort of corporate research into the causes of disease will shortly be brought to the touchstone of experiment, and we need hardly say that it is an experiment to which we heartily wish every possible success. The fund for the investigation of the nature, causes, and cure of cancer has at last been fairly established; and, although the resources at the command of the promoters have not as yet reached the amount which they desired to obtain, it has been wisely determined that operations should be commenced without delay. The Presidency of the Fund has been accepted by the Prince of Wales, with Lord Lister, Lord Strathcona, Mr. Balfour, Sir Frederic Bramwell, Sir William Broadbent, and Mr. Bischoffsheim as Vice-Presidents, an office which it is hoped will also be accepted by the Duke of Bedford and Mr. Wernher. Five trustees have been appointed: Sir Douglas Powell, by the College of Physicians; Sir Alfred Cooper, by the College of Surgeons; Dr. Whipham, Mr. Mullins, and Mr. Neumann, by donors of £1,000 or upwards; and Mr. Henry Morris, who has been indefatigable as a promoter of the undertaking, has been elected honorary treasurer, in which capacity he has received, or is immediately promised, subscriptions and donations reaching a total of £36,500. The function of the president, vice-presidents, and trustees will be largely to furnish an official body to which the actual proceedings of those who are engaged in the work of research can be reported from time to time; and the task of directing, sanctioning, and harmonising this work will devolve upon an executive committee, consisting of Sir William Broadbent, Sir William Church, Sir Henry Howse, Drs. Sydney Martin, Pye Smith, and Rose Bradford, Professor Sims Woodhead, and Messrs. Butlin, Watson Cheyne, Langton, McFadyean, and Henry Morris. This committee is now engaged in the settlement of preliminary arrangements, and the appointment of the persons to whom the actual investigations will in the first instance be committed.

The one consideration which gives pause to the hopefulness with which the proceedings of such a committee would naturally be regarded, is chiefly derived from the history of discovery in the past, and from the eminent and conspicuous individuality of the great majority of the men by whom, in every department of human activity, any permanent addition to knowledge has been made. In science, in the arts, in organisation, in achievement, it has everywhere been the man (in Carlylean phrase, the hero) and nowhere the corporation or the community that has been effective. A committee of the most respectable and accomplished potters of the sixteenth century would have pronounced Bernard Palissy to be a hopeless lunatic; and a committee of Aldershot generals would certainly have scoffed at De la Rey's narrow trench in front of the ridge at Magersfontein. Great discoverers in physiology, in medicine, or in surgery, from Harvey to Lord Lister, have usually had to force their way to the establishment of new truth against the opposition of the very people from whose ranks committees representative of the sciences in question would have been selected by their contemporaries. New truth, by reason of its newness, is apt to jar upon minds which are unprepared to receive it; and the division of responsibility in a committee is almost equally exposed to lead to a sanction of fruitless labour in a direction in harmony with the *idola theatri* of the time, or to a refusal of assistance in some direction which appears unpromising, but in which, nevertheless, the truth may ultimately be discovered. We can hardly conceive a more difficult question, for the very committee which we are considering, than that of the amount of time, of money, and of labour which it might be desirable to expend upon an investigation of Mr. Hutchinson's recent suggestion of the possibility of a connection between the spread of cancer and the wide diffusion of arsenic. The force of an original suggestion often resides in the fact that the person to whose mind it occurs, and who determines to follow it to a conclusion, is sustained by an incapacity of recognising defeat, and so perseveres, in the face of every discouragement and every difficulty, to the time of ultimate victory or failure. Intense faith of this description is not to be expected from a committee; which must therefore always be exposed to the risk of adventuring nothing, and so of gaining nothing. We do not put forward these considerations with a view to any disparagement either of the cancer committee itself or of the work in which it will be engaged, but solely because we think that the difficulties of that work should be as much appreciated as the benefits which will arise from its being carried to a successful issue. Nothing could be more deplorable than any manifestation, on the part of the donors of money or of the public, of ignorant impatience at the slow attainment or at the doubtful character of the results. It is manifest that the system of "corporate investigation," if once successfully applied to the solution of a great physiological problem, will become one of vast and indefinite value and importance, and it is hence eminently necessary to secure, as far as may be possible, that the first attempt in this direction shall not be rendered futile by any want of appreciation of the conditions essential to its conduct.

